# Machine learning-guided leaf margin classification and discovery that *BcPHB* promoter selection rewires a BcPHB–BcLMI1 module to shape leaf margins in *Brassica campestris*

**DOI:** 10.1186/s43897-026-00251-w

**Published:** 2026-10-13

**Authors:** Jingwen Chen, Airong Li, Tianqi Wu, Zhenyu Huang, Xiyao Li, Shan Xu, Yujie Wu, Yuan Tian, Weiwei Chen, Xinhua Quan, Xiangtan Yao, Sue Lin, Xuping Feng, Li Huang

**Affiliations:** 1 Laboratory of Cell & Molecular Biology, Institute of Vegetable Science, Zhejiang University, Hangzhou, 310058 China; 2 College of Biosystems Engineering and Food Science, Zhejiang University, Hangzhou, 310058 China; 3 Zhejiang Provincial Key Laboratory of Wetland Intelligent Monitoring and Ecological Restoration, Zhejiang-Canada Joint Laboratory on Biodiversity and Global Change, College of Life and Environmental Sciences, Hangzhou Normal University, Hangzhou, 310036 China; 4 Jiaxing Academy of Agricultural Sciences, Jiaxing, 314000 China; 5 Institute of Life Sciences, Wenzhou University, Wenzhou, 325000 China; 6 Hainan Institute of Zhejiang University, Sanya, 572025 China

**Keywords:** Digital phenotyping, Leaf development, HD-ZIP transcription factor, Domestication, Morphological diversification

## Abstract

The morphological diversification of leaf margins reflects the evolutionary reshaping of conserved developmental programs. However, fully deciphering the underlying molecular basis remains challenging due to difficulties in phenotype quantification and the incomplete characterization of complex regulatory networks. In this study, we established a computer-vision framework to decode leaf lobe trait and identified the HD-ZIP III factor *PHABULOSA* (*BcPHB*), combining genome-wide association studies (GWAS) and selective sweep analysis in *Brassica campestris*. We found that promoter variation defines two major haplotypes, *BcPHB*^*HapI*^ and *BcPHB*^*HapII*^, driving divergent expression levels associated with entire and lobed leaves. Functionally, *BcPHB* knockdown lines (*BcPHB*^*ami*^) exhibited lobed margins, whereas complementation of lobed accessions with the *BcPHB*^*HapI*^ promoter partially restored the entire-leaf phenotype. Similarly, overexpression of *LATE MERISTEM IDENTITY 1* (*BcLMI1*), a conserved regulator of leaf lobation, phenocopied the *BcPHB*^*ami*^ lines. Mechanistically, *BcPHB* and *BcLMI1* form a reciprocal negative feedback loop that stabilizes margin patterning. Furthermore, domestication-driven selection of *BcPHB*^*HapI*^ facilitated the transition from ancestral lobed leaves to cultivated entire forms. This study reveals a pivotal developmental module and illustrates how regulatory evolution drives morphological diversification in crops.

**Supplementary Information:**

The online version contains supplementary material available at https://doi.org/10.1186/s43897-026-00251-w.

## Core

This study integrates machine-learning phenotyping with GWAS and selective sweep analysis to resolve leaf margin evolution in *Brassica campestris*. We identify a BcPHB-BcLMI1 reciprocal feedback loop as a key model of margin patterning and demonstrate that *cis*-regulatory selection in the *BcPHB* promoter drove the domestication-mediated transition from lobed to entire leaves. These findings provide a generalizable framework for understanding how regulatory rewiring orchestrates crop morphological diversification.

## Gene & accession numbers

Sequence data reported in this article can be retrieved from the Brassicaceae Database (BRAD) (http://www.brassicadb.cn/) under the accession numbers: *BcPHB* (BraA04g025970.3.5C) and *BcLMI1* (BraA10g032390.3.5C).

## Introduction

Plant leaf morphology, critical for light capture, gas exchange, and carbon assimilation, dictates plant architecture, stress resilience, and productivity (Huang et al. 2015; Lei et al. 2024). Precise genetic regulation of leaf margin forms (entire, serrated, lobed) is vital for designing ideal plant architectures (IPA) in modern crop improvement. This morphological diversity, reflecting evolutionary adaptations, is crucial for botanical research and crop genetic enhancement.

Within the Brassicaceae family, leaf margin diversity is particularly pronounced. *Brassica campestris* syn. *B. rapa*, a major leafy vegetable, exemplifies this variation with subspecies showing entire, serrated, and highly complex lobed patterns (Kawakatsu et al. 2021). Such lobed architectures can confer significant agronomic advantages, such as enhanced photosynthetic efficiency in high-density plantings (Heng et al. 2020; Semchenko and Zobel 2007) and improve shade response (Vogel 2009).

However, our ability to phenotype these intricate traits has been persistently hindered by a critical bottleneck: the lack of objective and high-throughput phenotyping methods. Traditional approaches rely on subjective visual classification, which fails to capture the continuous spectrum of variation in leaf shape. While computer vision and machine learning have been applied to quantify basic plant traits in *B. campestris* (Li et al. 2023c; Xu et al. 2024; Yang et al. 2024; Jin et al. 2025), their application to derive a robust, quantitative index that represents the degree of leaf lobation remains a significant challenge. Therefore, developing a precise, automated framework to convert complex leaf morphology into a standardized numerical value is an essential step for this key agronomic trait.

Concurrently, the architecture of the molecular networks governing leaf margin development remains incompletely understood. Specifically, although the HD-ZIP I family member *LATE MERISTEM IDENTITY1* (*LMI1*)/*REDUCED COMPLEXITY* (*RCO*) has been established as a conserved core module controlling leaf lobation across diverse species, including *Cucurbita pepo* (Bo et al. 2022), *Gossypium hirsutum* (Andres et al. 2017; Chang et al. 2019)*, Medicago truncatula* (Wang et al. 2021), and crucifers such as *Cardamine hirsuta* (Vlad et al. 2014b), and various *Brassica* crops (Heng et al. 2020; Hu et al. 2018; Li et al. 2023a, 2023b; Ni et al. 2017; Zhang et al. 2021), the upstream regulatory mechanisms that modulate its expression are still poorly defined.

Fundamentally, leaf margin patterning is not an isolated process; it is embedded within the overall program of leaf morphogenesis. A cornerstone of this program is the establishment of the adaxial-abaxial polarity axis, which is classically governed by the HD-ZIP III family of transcription factors, including *PHABULOSA* (*PHB*) (McConnell and Barton 1998; McConnell et al. 2001; Sebastian et al. 2015). To date, studies on *PHB* have focused on its fundamental role in establishing the primary axis; severe loss-of-function mutants display extreme phenotypes like leaf radialization (Golz 2006; McConnell et al. 2001; Rong et al. 2019). However, such severe phenotypes may mask the potential finer roles for *PHB* in quantitatively modulating the final leaf morphology, such as the margin. Given PHB’s status as a fundamental developmental regulator that governs the primary leaf axis, its potential involvement in refining the downstream patterning of the leaf margin represents a critical and unexplored link in understanding leaf morphogenesis.

In this study, we first established a pipeline for precise leaf lobation quantification using computer vision and machine learning. In parallel, to dissect the molecular mechanisms governing leaf margin diversity, we focused on the upstream regulation of *BcLMI1*, a known pivotal gene in leaf morphogenesis. We identified BcPHB as a critical upstream regulator that patterns the leaf margin by modulating *BcLMI1* expression. Furthermore, we demonstrated that this regulatory function is rooted in distinct haplotypes within the promoter of *BcPHB*, which drive its divergent expression levels. Collectively, our work uncovers a key regulatory module that links the establishment of adaxial-abaxial polarity to the final patterning of the leaf margin, providing a mechanistic framework for the evolution of plant organ form.

## Results

### High-throughput digital phenotyping decodes the complexity of leaf margins

**Fig. 1:**
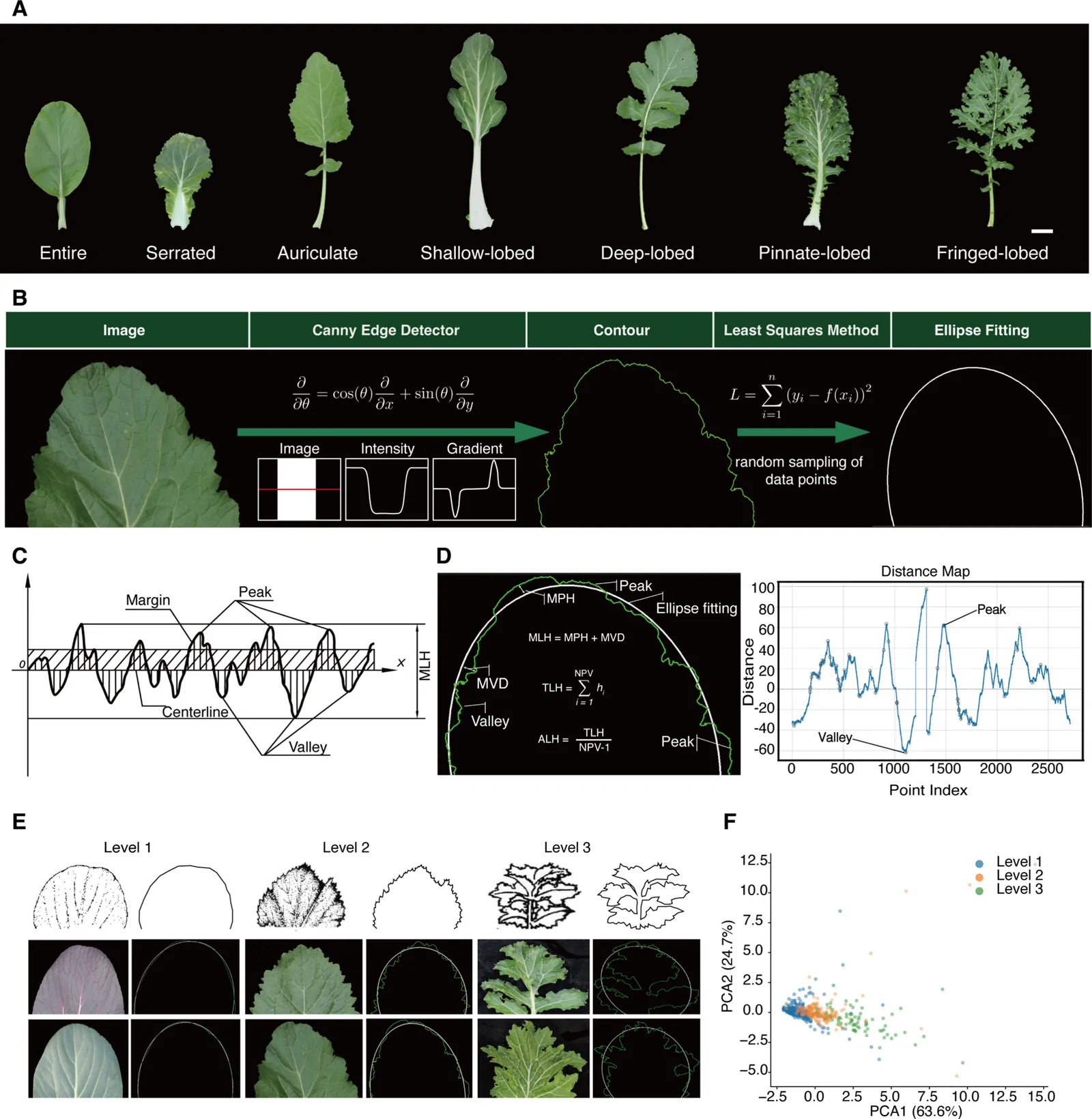
Digital phenotyping pipeline for quantifying leaf lobation in *Brassica campestris*. **A** Representative images showing the spectrum of leaf margin variation in *B. campestris*, from entire (left) to fringed-lobed (right). **B** Image-processing workflow for extracting the leaf contour from RGB images. **C** Quantification of leaf-lobation deviation using a fitted reference ellipse: contour points outside the ellipse are defined as lobe peaks, whereas points inside the ellipse are defined as sinus valleys. **D** Schematic definition of the six contour-derived lobation metrics: Maximum Peak Height (MPH), Maximum Valley Depth (MVD), Maximum Lobing Height (MLH), Number of Peaks and Valleys (NPV), Total Lobing Height (TLH), and Average Lobing Height (ALH). **E** Representative leaf morphotypes (Level 1–3) across germplasm resources. **F** Clustering of accessions based on the combination of six key metrics

To overcome the limitations of subjective, qualitative classification of *B*. *campestris* leaf lobation (Fig. 1A), the pipeline first extracted the leaf contour from RGB images using edge detection and morphological filtering (Fig. 1B). A reference ellipse was then fitted to the leaf contour to provide a standardized baseline for lobation measurement (Fig. 1C).

Points on the contour lying outside the ellipse were defined as peaks, while points inside were defined as valleys. Based on the signed deviations between the contour and the reference ellipse, contour-derived metrics were calculated to describe leaf lobation complexity: Maximum Peak Height (MPH), Maximum Valley Depth (MVD), Maximum Lobing Height (MLH), Number of Peaks and Valleys (NPV), Total Lobing Height (TLH), and Average Lobing Height (ALH) (Fig. 1D, Table S1).

To validate the descriptive power of these metrics, we trained distinct machine learning models (k-nearest neighbors [KNN], Naïve Bayes [NB], random forest [RF], support vector machine [SVM], and a neural network [NN]) to classify leaves into three lobing levels based solely on these quantitative inputs. Principal Component Analysis (PCA) showed partial separation of the different leaf morphology classes (Fig. 1E, F). Because 2D projections may understate class separability, we further evaluated all five classifiers using complementary metrics in the original feature space. Under stratified fivefold cross-validation, the classifiers showed robust but metric-dependent performance in distinguishing the three annotated lobation levels (Fig. S1), visually confirming the reliability and discriminative power of our phenotyping framework.

### Natural variation in the *BcPHB* promoter associates with leaf lobation

**Fig. 2:**
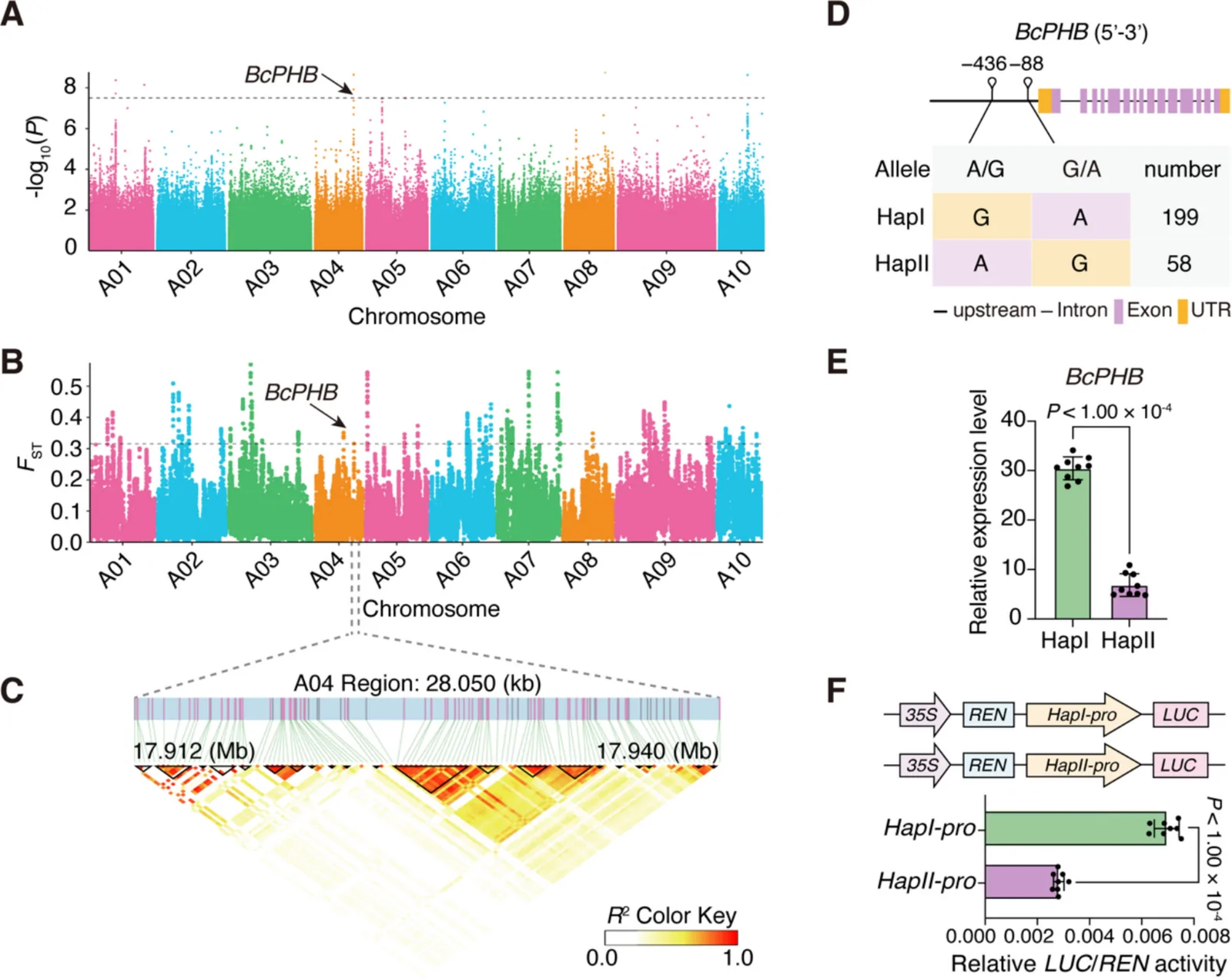
Identification and analysis of major leaf lobe loci in *Brassica campestris*. **A** Manhattan plot for genome-wide association study (GWAS) using the phenotype data of 291 accessions containing different leaf margin types. The horizontal dashed line indicates the threshold (0.05/total SNPs, − log_10_(*P*-value) = 7.50). The black arrow points to the significant signals of *BcPHB*. A01-A10 represents the ten chromosomes of *B. campestris*. **B** Selective-sweep analysis between the lobed leaf and entire leaf accessions. The *F*_ST_ values are plotted against the position on each chromosome (A01-A10). The horizontal dashed line indicates the genome-wide threshold (Top 1%, *F*_ST_ > 0.32). The black arrow points to the significant signals of *BcPHB*. **C** Linkage disequilibrium analysis of SNPs surrounding *BcPHB.* A04: The chromosome 4 of *B. campestris*. **D** Haplotype distribution of *BcPHB* (*n* > 10 per haplotype). Gene structure (top) and SNP variations (bottom) are shown, Hap: Haplotype. Heterozygous accessions (*n* = 34) were not included in the figure. **E** Comparison of the *BcPHB* transcript level between randomly selected accessions of HapI and HapII, respectively. Relative expression levels were normalized to the gene *BcUBC10*. The error bar represents the means ± SDs (*n* = 9). **F** Schematic diagrams of the constructs used for the dual-luciferase assay. Promoter activity analysis of the promoter of the entire leaf accession (HapI) and the promoter of the lobed leaf accession (HapII). The error bars are means ± SDs (*n* = 8), Welch’s* t*-test. 35S: *Cauliflower mosaic virus* 35S promoter, REN: Renilla luciferase, LUC: Luciferase

To investigate the genetic basis of leaf margin changes during *B. campestris* domestication, we analyzed resequencing data from our core germplasm collection containing 291 accessions. The GWAS analysis identified 40 genes related to this trait (Fig. 2A, Fig. S2, Table S2). Additionally, selective sweep analysis revealed 776 genes (Fig. 2B, Table S3). Only four genes (BraA04g025970.3.5C, BraA04g025980.3.5C, BraA04g025990.3.5C, and BraA04g026000.3.5C) within a 28.050 kb interval block (spanning 17.912 to 17.940 Mb on chromosome 4) were identified by both methodologies (Fig. 2C, Table S4). Among these, only BraA04g025970.3.5C has previously been annotated to leaf development and was therefore selected for further investigation (McConnell et al. 2001; Mallory et al. 2004; Prigge et al. 2005) (Table S5). This gene shares high homology with the Arabidopsis *PHB* gene and was named *BcPHB*. Then, we identified two major haplotypes, HapI and HapII, defined by two key single-nucleotide polymorphisms (SNPs) within the promoter of *BcPHB* (Fig. 2D, Table S6). These haplotypes showed an association with leaf margin morphology: HapI was almost exclusively found in entire-leaf accessions, while HapII was predominant in lobed-leaf accessions (Fig. 2D).

Analysis of transcript levels confirmed the functional consequence of this divergence. Accessions carrying the entire-leaf HapI exhibited significantly higher *BcPHB* expression compared to those with the lobed-leaf HapII (Fig. 2E). To directly test if these promoter variations cause the expression difference, we performed promoter activity assays. As hypothesized, the HapI promoter drove substantially higher reporter gene activity than the HapII promoter (Fig. 2F). Reverting these SNPs in the HapII background (*proBcPHB*^*HapII*^*-rev*) restored promoter activity to HapI levels, whereas reciprocal mutations in the HapI background (*proBcPHB*^*HapI*^*-mut*) significantly abolished its high activity (Fig. S3). These results demonstrate that the identified SNPs are functionally causative for the differential *BcPHB* expression.

### *BcPHB* functions as a key suppressor of leaf lobation

**Fig. 3:**
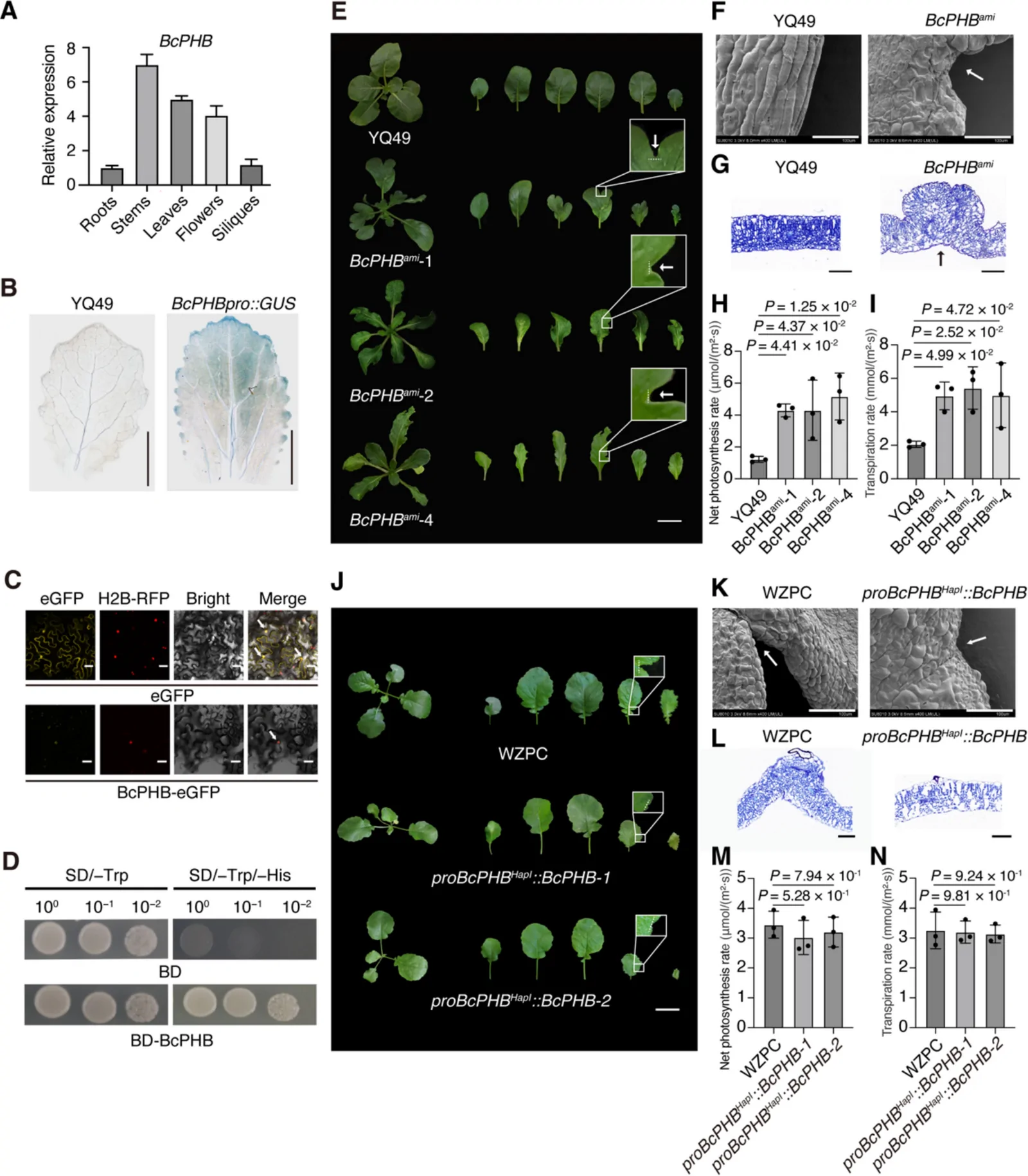
Functional characterization of *BcPHB* in leaf development. **A** Expression level of *BcPHB* in different tissues of *Brassica campestris* (roots, stems, leaves, flowers, and siliques). Relative expression levels were normalized to the gene *BcUBC10*. The error bar represents the means with SDs. **B** GUS-staining of the 7 days after sowing (DAS), first true leaf of YQ49 (entire-leaf) and *BcPHBpro::GUS* plant. Scale bars = 5 mm. Pro: promoter, GUS: Glucuronidase. **C** The subcellular localization of BcPHB in the *Nicotiana benthamiana* leaves, together with the nuclear marker construct H2B-red fluorescent protein (RFP). *35S::eGFP* was used as a control. Scale bars = 50 μm. 35S: *Cauliflower mosaic virus* 35S promoter, eGFP: enhanced green fluorescent protein. **D** Transactivation assay of BcPHB in yeast cells. The empty vector pGBKT7 (BD) was used as the negative control. Trp: tryptophan, His: histidine. **E**, **J** YQ49 (entire-leaf) and *BcPHB*^*ami*^ plants at 35 days after sowing (DAS) (**E**), WZPC (lobed-leaf) and *proBcPHB*^*HapI*^*::BcPHB* plants at 25 DAS (**J**). Scale bar = 4 cm. A magnified view of the leaf margin is shown in the white box. **F**, **K** Scanning electron microscopy (SEM) images of the adaxial epidermis of the same part of the leaf in YQ49 and *BcPHB*^*ami*^ plants (**F**), WZPC (lobed-leaf) and *proBcPHB*^*HapI*^*::BcPHB* plants (**K**), scale bars = 100 μm. The white arrow points to the depression of the leaf margin. **G**, **L** Cross-section of the same part of the leaf in YQ49 and *BcPHB*^*ami*^ plants (**G**), WZPC and *proBcPHB*^*HapI*^*::BcPHB* plants (**L**). Scale bars = 100 μm. The black arrow points to the depression of the leaf margin. **H**, **M** Net photosynthesis rate measurements of YQ49 and *BcPHB*^*ami*^ plants (**H**), WZPC and *proBcPHB*^*HapI*^*::BcPHB* plants (**M**). **I**, **N** Transpiration rate measurements of YQ49 and *BcPHB*^*ami*^ plants (**I**), WZPC and *proBcPHB*^*HapI*^*::BcPHB* plants (**N**). Measurements were repeated three times for each plant; the error bar represented the means ± SDs (*n* = 3), one-way ANOVA, and Dunnett’s test. Ami: artificial microRNA

To investigate the function of *BcPHB*, we first characterized its expression profile in an entire-leaf *B. campestris* cultivar. *BcPHB* was expressed throughout the plant, with the highest levels detected in stems, followed by leaves, with lower expression in flowers, siliques, and roots (Fig. 3A). Expression analysis across different leaf positions revealed higher *BcPHB* levels in the 1 st to 3rd true leaves, followed by a decline in the 4th to 6th leaves (Fig. S4). Histochemical staining of transgenic plants harboring a GUS reporter driven by the HapI promoter exhibited strong GUS activity detected in leaf margins and vascular tissues (Fig. 3B). To determine its subcellular localization, 35S::BcPHB-eGFP was transiently expressed in *Nicotiana benthamiana* leaves alongside the nuclear marker H2B‐red fluorescent protein (RFP). BcPHB-eGFP signals co-localized with H2B-RFP, confirming nuclear localization of BcPHB (Fig. 3C). Further transactivation assays demonstrated the transcriptional activity of BcPHB (Fig. 3D).

To explore the function of BcPHB in leaf development, we generated *BcPHB* knockdown transgenic plants (*BcPHB*^*ami*^) using an artificial microRNA (amiRNA) approach in an entire-leaf *B*. *campestris* background (YQ49). RT-qPCR analysis confirmed significantly reduced *BcPHB* transcript levels in *BcPHB*^*ami*^ leaves compared with YQ49 plants (Fig. S5). *BcPHB*^*ami*^ plants exhibited shallow-lobed leaf margins, in contrast to the entire margins observed in YQ49 plants (Fig. 3E). This morphological change was accompanied by underlying anatomical alterations, including irregularly shaped epidermal cells and disorganized mesophyll tissues at the leaf margin (Fig. 3F, G). *BcPHB*^*ami*^ plants also exhibited significantly elevated net photosynthesis and transpiration rates compared to YQ49 (Fig. 3H, I), demonstrating that *BcPHB* suppression has consequences at both the morphological and physiological levels. Conversely, when we drove *BcPHB* expression using its native high-expression self-promoter (*proBcPHB*^*HapI*^::*BcPHB*) in a deep-lobed *B. campestris* background (WZPC), the native lobed phenotype was suppressed, leading to a much smoother leaf margin (Fig. 3J-L, Fig. S6), while the photosynthetic and transpiration rates remained comparable to the WZPC (Fig. 3M, N).

### BcPHB patterns leaf margins by acting as the direct upstream repressor of *BcLMI1*

**Fig. 4:**
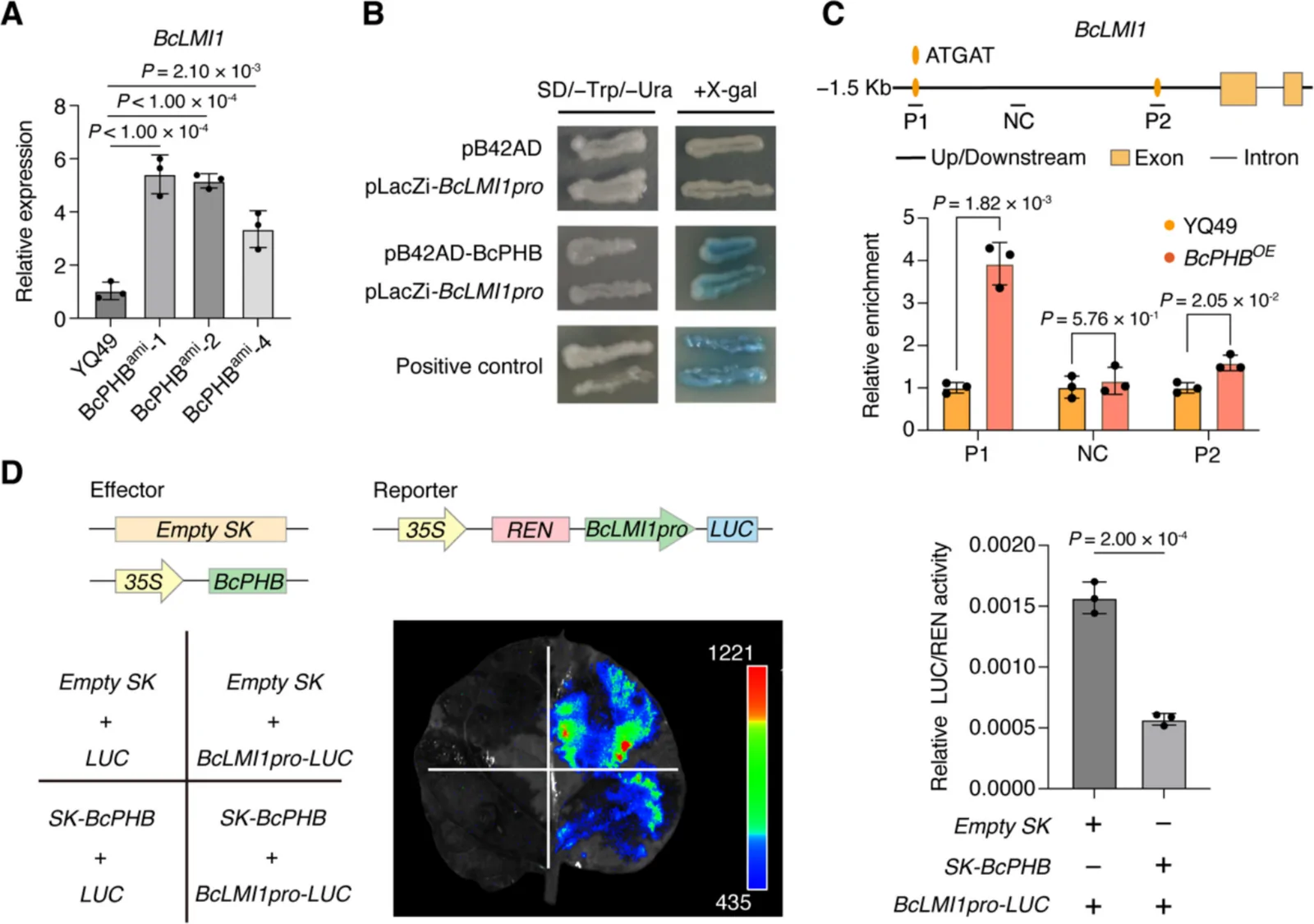
BcPHB directly represses *BcLMI1*. **A** Relative expression level of *BcLMI1* in the leaves of *BcPHB*^*ami*^ transgenic plants. Relative expression levels were normalized to the gene *BcUBC10*. Data are means ± SDs (*n* = 3). One-way ANOVA Dunnett’s test. **B** Yeast-one-hybrid assays (Y1H) show the binding of BcPHB to the *BcLMI1* promoter. The EGY48 yeast strain YS3021 (Coolaber) was used as the positive control. Trp: Tryptophan, Ura: Uracil. X-gal: 5-Bromo-4-chloro-3-indolyl *β*-D-galactoside. **C** Chromatin immunoprecipitation quantitative polymerase chain reaction (ChIP-qPCR) assay of BcPHB binding to the *BcLMI1* promoter in YQ49 plants and *BcPHB*^*OE*^ transgenic plants (with *BcPHB* fused to 3 × HA tag). A monoclonal anti-HA antibody was used. Fold enrichment was quantified by normalizing the immunoprecipitation signal in transgenic lines to that in YQ49. Data are means ± SDs (*n* = 3); statistical significance was determined by Student’s* t*-test. P1 and P2 represent the predicted motifs (ATGAT) bound by BcPHB, NC: negative control, represents the fragment that could not bind by BcPHB. **D** The repressive regulation of *BcLMI1* by BcPHB was detected using a dual-luciferase (DLR) assay. Luciferase/Renilla (LUC/REN) ratio was low in *Nicotiana benthamiana* leaves when co-expressing SK-BcPHB and *BcLMI1pro-LUC*. The empty pGreenII-62-SK (SK) vector was used as a negative control. Error bars represent means ± SDs (*n* = 3). *P*-values indicate statistically significant differences determined by Student’s *t*-test. 35S: *Cauliflower mosaic virus* 35S promoter, REN: Renilla luciferase, LUC: Luciferase

Our genetic analyses identified *BcPHB* as the key causal gene for leaf margin variation in *B. campestris*. Interestingly, *BcPHB* and BcLMI1 both belong to the HD-ZIP transcription factor family (Fig. S7), a previously reported, conserved regulator known to promote leaf margin lobation among eudicots (Fig. S8). To investigate whether *BcPHB* exerts its function by modulating this known gene, we set out to clarify the regulatory relationship between them. *BcLMI1* transcript levels were significantly higher in lobed-leaf accessions than in entire-leaf accessions (Fig. S9), and were significantly upregulated in *BcPHB*^*ami*^ plants compared with YQ49 plants (Fig. 4A), suggesting that BcPHB negatively regulates *BcLMI1* expression. A yeast one-hybrid (Y1H) assay confirmed that BcPHB directly binds to the *BcLMI1* promoter (Fig. 4B). As PHB transcription factors recognize and bind ATGAT motifs within DNA sequences (Bertolotti et al. 2021), which are present in the *BcLMI1* promoter (Fig. 4C), we performed a chromatin immunoprecipitation quantitative PCR (ChIP-qPCR) assay using the *BcPHB*^*OE*^-HA transgenic plants with overexpression of *BcPHB* and the YQ49 plants (Fig. 4C; Fig. S10). Significant enrichment of BcPHB in the *BcLMI1* promoter region in *BcPHB*^*OE*^ plants confirmed direct binding of BcPHB to the *BcLMI1* promoter. To assess whether BcPHB binding represses *BcLMI1* transcription, we conducted a luciferase (LUC) bioluminescence imaging assay and a dual-luciferase reporter (DLR) assay using a reporter construct containing the *BcLMI1* promoter fused to the firefly luciferase gene and an effector construct with BcPHB driven by the CaMV35S promoter (Fig. 4D). Upon co-infiltration of the reporter and BcPHB effector into *N. benthamiana* leaves, luciferase bioluminescence intensity, and the firefly luciferase/Renilla luciferase (LUC/REN) ratio were significantly reduced compared with the control (co-infiltration of the reporter and empty SK effector) (Fig. 4D).

### BcLMI1 is essential for promoting leaf lobation

**Fig. 5:**
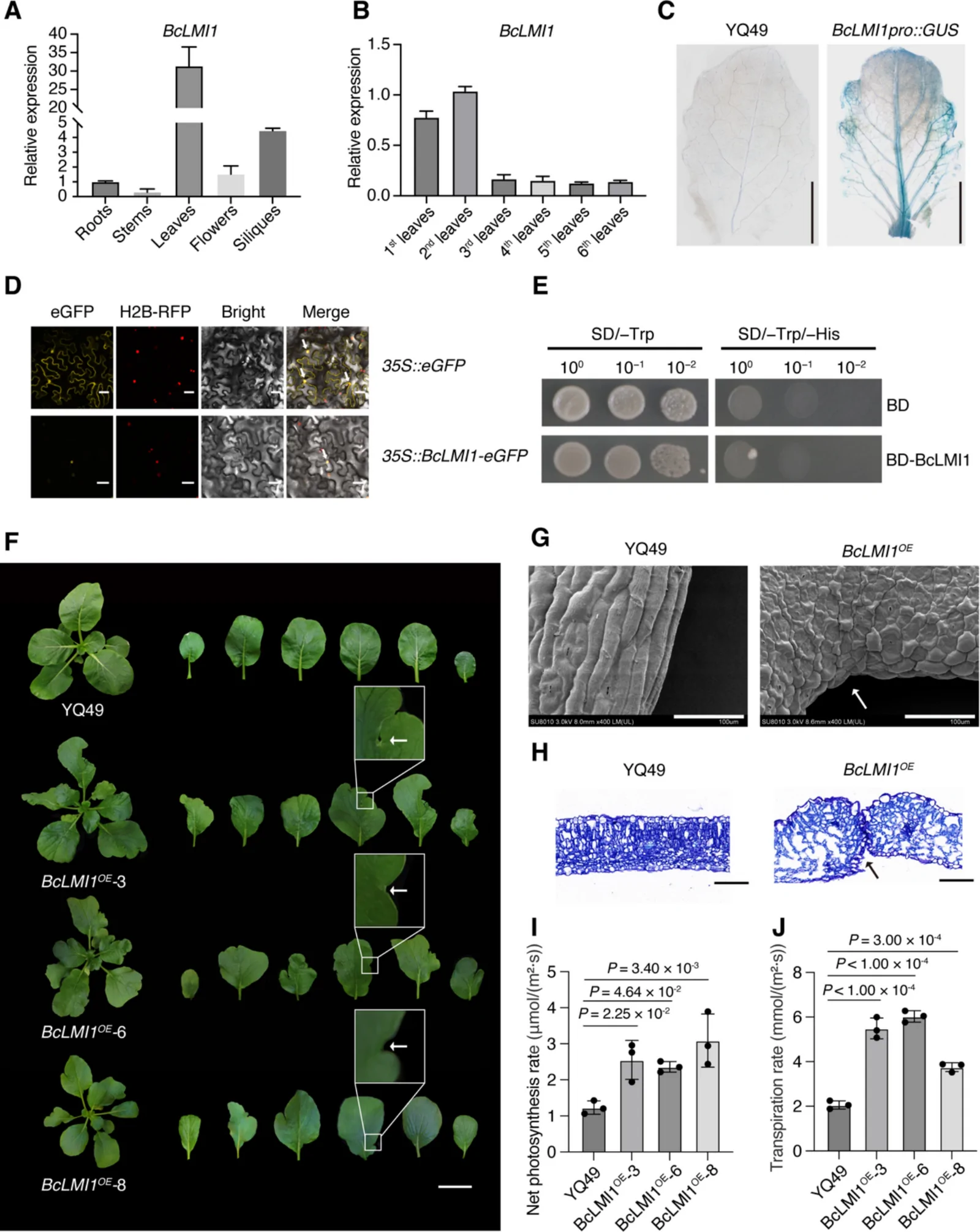
Expression pattern and gene function of *BcLMI1*. **A** Expression level of *BcLMI1* in different tissues of *Brassica campestris* (roots, stems, leaves, flowers, and siliques). Relative expression levels were normalized to the gene *BcUBC10*. The error bar represents the means with SDs. **B** The expression level of *BcLMI1* in leaves at different leaf positions of *B. campestris* (1–6 represents the first to the sixth true leaves). Relative expression levels were normalized to the gene *BcUBC10*. The error bar represents the means with SDs. **C** GUS-staining of the 7 days after sowing (DAS), first true leaf of YQ49 (entire-leaf) plant, and *BcLMI1pro::GUS* transgenic plant. Scale bars = 5 mm. Pro: promoter, GUS: Glucuronidase. **D** The subcellular localization of BcLMI1 in the *Nicotiana benthamiana* leaves, together with the nuclear marker construct H2B-red fluorescent protein (RFP). *35S::eGFP* was used as a control. Scale bars = 50 μm. 35S: *Cauliflower mosaic virus* 35S promoter, eGFP: enhanced green fluorescent protein. For convenience of comparison, the photographs of 35S::eGFP used in (**D**) as control are identical to those in Fig. 3C. **E** Transactivation assay of BcLMI1 in yeast cells. The empty vector pGBKT7 (BD) was used as the negative control. Trp: tryptophan, His: histidine. **F** YQ49 (entire-leaf) and *BcLMI1*^*OE*^ plants at 35 days after sowing (DAS). Scale bar = 4 cm. A magnified view of the leaf margin is shown in the white box. **G** Scanning electron microscopy (SEM) images of the adaxial epidermis of the same part of the leaf in the YQ49 and *BcLMI1*^*OE*^ plant, scale bars = 100 μm. The white arrow points to the depression of the leaf margin. **H** Cross-section of the same part of the leaf in the YQ49 and *BcLMI1*^*OE*^ plant. Scale bars = 100 μm. The black arrow points to the depression of the leaf margin. For convenience of comparison, the photographs of YQ49 used in (**F**), **G**, and (**H**) as control are identical to those in Fig. 3E, F, G, respectively. **I** Net photosynthesis rate measurements and (**J**) Transpiration rate measurements of YQ49 and *BcLMI1*^*OE*^ plants. Measurements were repeated three times for each plant; the error bar represented the means ± SDs (*n* = 3), one-way ANOVA, and Dunnett’s test. The arrows point to the depression of the leaf margin

To further validate the role of *BcLMI1* in leaf lobe development, RT-qPCR was first performed to detect its expression patterns. In an accession with entire leaves, *BcLMI1* was highly expressed in leaves, followed by siliques, flowers, roots, and stems (Fig. 5A). In the leaves, its expression was highest in the 1 st and 2nd true leaves and declined in the 3rd to 6th true leaves (Fig. 5B). Histochemical staining of transgenic plants carrying a *BcLMI1* promoter-driven GUS construct revealed strong GUS activity in vascular bundles and along leaf margins (Fig. 5C). Co-expression of a *35S::BcLMI1-eGFP* vector with the nuclear marker H2B-RFP in leaf epidermal cells confirmed the localization of BcLMI1 in cell nuclei (Fig. 5D). Transactivation assays were then performed to assess the transcriptional activation potential of BcLMI1 in vitro. Y2H Gold yeast strains expressing either the negative control pGBKT7 (BD) or BD-BcLMI1 failed to grow on SD/-Trp/-His selective medium (Fig. 5E), indicating that BcLMI1 lacks intrinsic transcriptional activation activity.

Then, transgenic overexpression plants of *BcLMI1* (*BcLMI1*^*OE*^) were generated in an entire-leaf *B*. *campestris* background (YQ49). Compared with YQ49 plants, three independent *BcLMI1*^*OE*^ lines exhibited significantly increased *BcLMI1* transcript levels (Fig. S11). *BcLMI1*^*OE*^ plants developed shallow-lobed leaves (Fig. 5F). SEM analysis revealed that epidermal cells at the leaf margin depressions of *BcLMI1*^*OE*^ plants were smaller, with spongy and palisade tissues near the leaf margin exhibiting distinct structural changes compared with YQ49 plants (Fig. 5G, H). Additionally, *BcLMI1*^*OE*^ plants displayed significantly higher net photosynthesis and transpiration rates than YQ49 plants (Fig. 5I, J). Collectively, these findings indicate that *BcLMI1* overexpression phenocopies the *BcPHB*^*ami*^ lines, demonstrating that BcLMI1 promotes leaf lobe development in *B. campestris.*

### A reciprocal BcPHB-BcLMI1 negative feedback loop stabilizes leaf margin morphogenesis

**Fig. 6:**
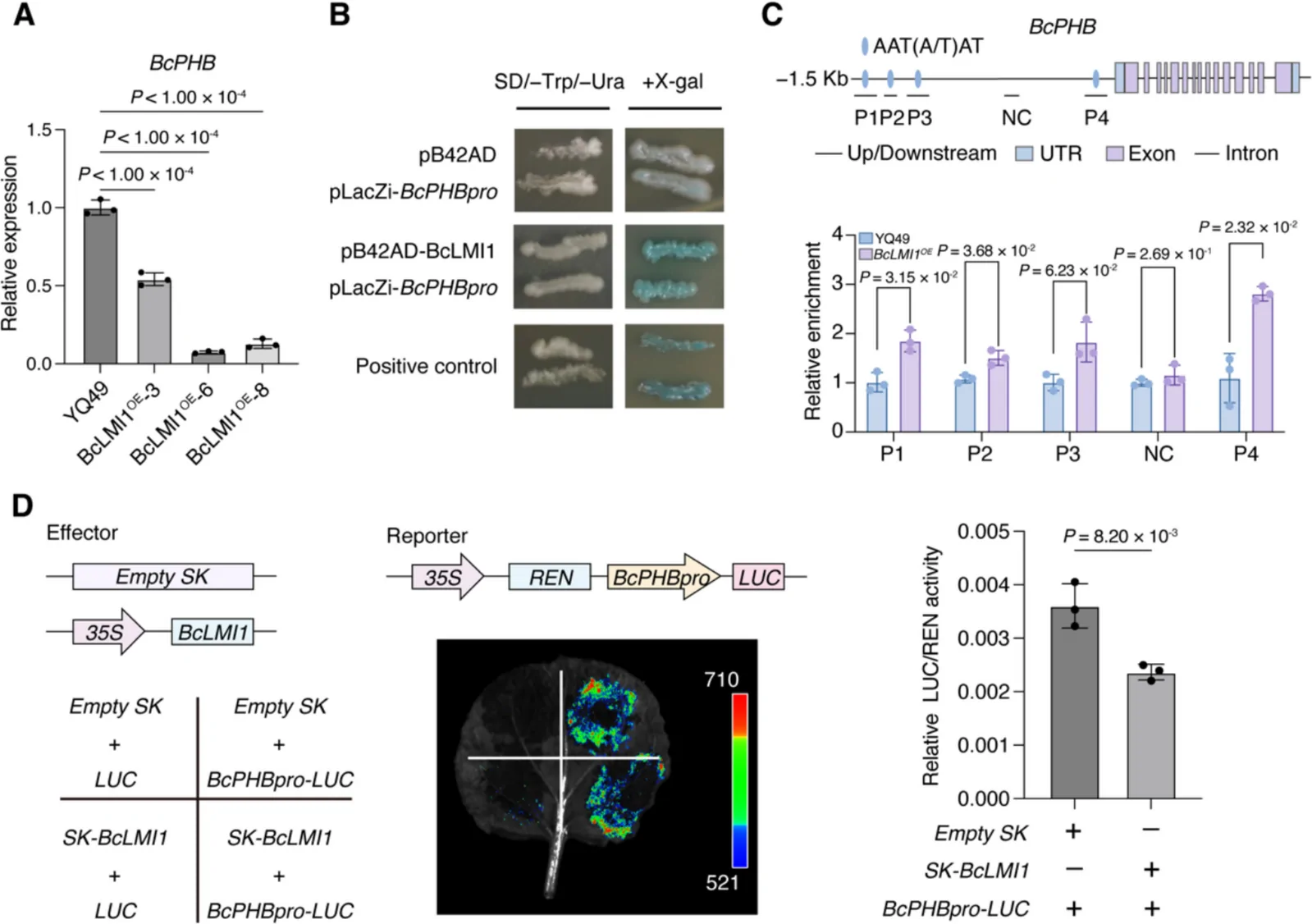
BcLMI1 causes leaf lobes through direct binding to the promoter of *BcPHB*. **A** Relative expression level of *BcPHB* in the leaves of *BcLMI1*^*OE*^ transgenic plants. Relative expression levels were normalized to the gene *BcUBC10*. Data are means ± SDs (*n* = 3). One-way ANOVA Dunnett’s test. **B** Yeast-one-hybrid assays (Y1H) show the binding of BcLMI1 to the *BcPHB* promoter. The EGY48 yeast strain YS3021 (Coolaber) was used as the positive control. Trp: tryptophan, Ura: uracil. X-gal: 5-Bromo-4-chloro-3-indolyl *β*-D-galactoside. **C** Chromatin immunoprecipitation quantitative polymerase chain reaction (ChIP-qPCR) assay of BcLMI1 binding to the *BcPHB* promoter in YQ49 and *BcLMI1*^*OE*^ plants (with BcLMI1 fused to 3 × HA tag). A monoclonal anti-HA antibody was used. Fold enrichment was quantified by normalizing the immunoprecipitation signal in transgenic lines to that in YQ49. Data are means ± SDs (*n* = 3); statistical significance was determined by Student’s *t*-test. P1, P2, P3, and P4 represent the predicted motifs (AAT(A/T)AT) bound by BcLMI1, NC: negative control, represents the fragment that could not bind by BcLMI1. **D** The repressive regulation of *BcPHB* by BcLMI1 was detected using a dual-luciferase (DLR) assay. Luciferase/Renilla (LUC/REN) ratio was low in *N. benthamiana* leaves when co-expressing SK-BcLMI1 and *BcPHBpro-LUC*. The empty pGreenII-62-SK (SK) vector was used as a negative control. Error bars represent means ± SDs (*n* = 3). *P*-values indicate statistically significant differences determined by Student’s *t*-test. 35S: *Cauliflower mosaic virus* 35S promoter, REN: Renilla luciferase, LUC: Luciferase

Previous studies have shown that the LMI1 protein binds to AAT(A/T)AT motifs in DNA sequences (Franco-Zorrilla et al. 2014; Vuolo et al. 2018), which are present in the promoter of *BcPHB*. Consistent with a potential regulatory relationship, *BcPHB* expression was significantly lower in *BcLMI1*^*OE*^ plants than in YQ49 plants (Fig. 6A). This prompted us to investigate whether BcLMI1 directly modulates *BcPHB* expression by binding to its promoter. To test this hypothesis, we performed a Y1H assay, which confirmed BcLMI1 binding to the *BcPHB* promoter (Fig. 6B). Additionally, ChIP-qPCR analysis revealed significant enrichment of BcLMI1 in the *BcPHB* promoter region in *BcLMI1*^*OE*^-HA plants, indicating in vivo binding between BcLMI1 and the *BcPHB* promoter (Fig. 6C). To determine whether BcLMI1 binding represses *BcPHB* transcription, we conducted a luciferase bioluminescence imaging assay and a DLR assay. Co-infiltration of BcLMI1 with the *BcPHBpro*-LUC construct resulted in a significant decrease in fluorescence intensity and the LUC/REN ratio (Fig. 6D), demonstrating the inhibitory effect of BcLMI1 on *BcPHB* promoter activity. These findings indicate that BcLMI1 directly represses *BcPHB* expression, forming a negative feedback loop that regulates leaf lobe development in *B. campestris*.

### Domestication favored *BcPHB* promoter alleles to optimize leaf architecture

**Fig. 7:**
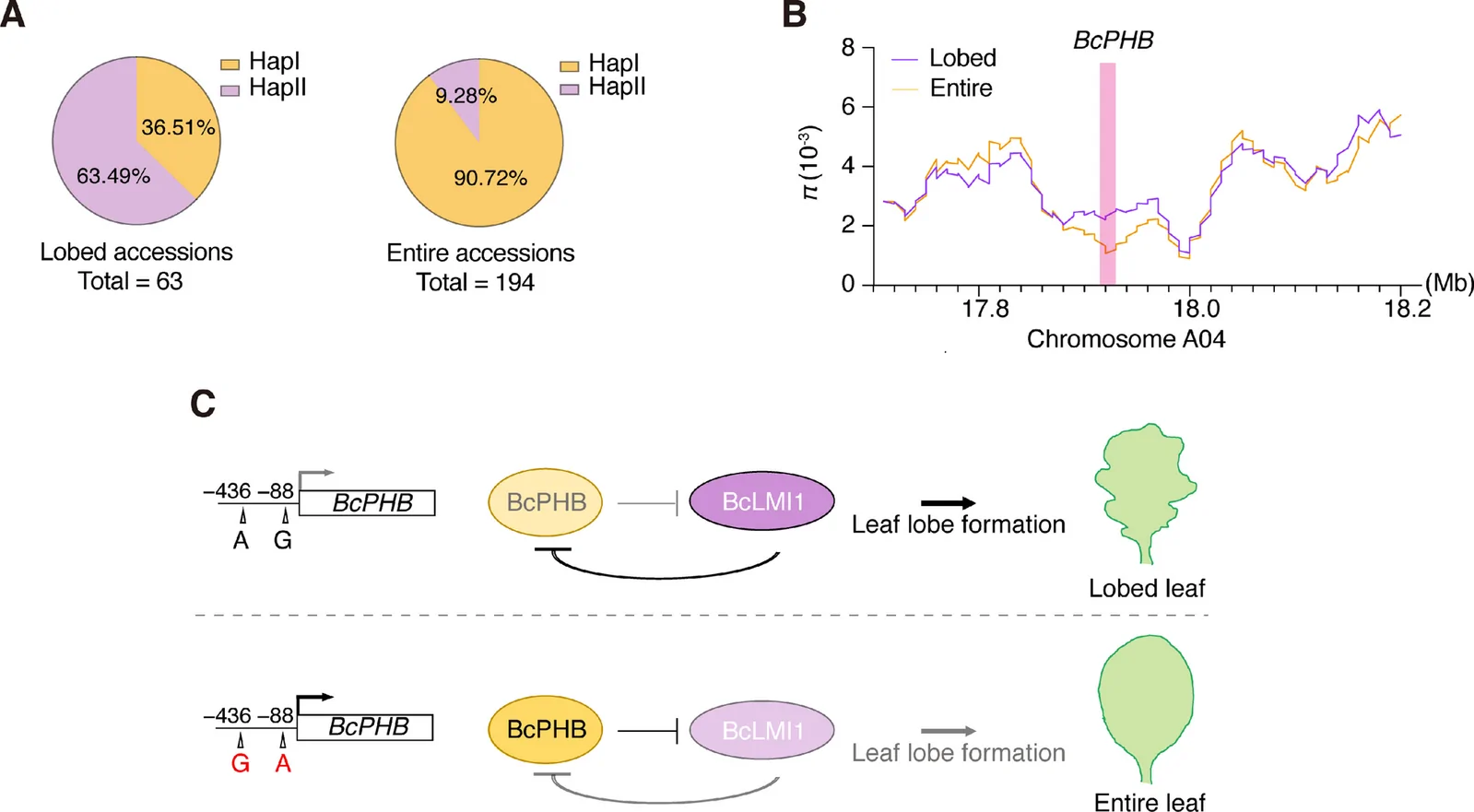
Genetic differentiation of *BcPHB* in *B. campestris* germplasm and a proposed working model of *BcPHB* in controlling leaf lobe formation in *B. campestris*. **A** Haplotype frequency distribution of *BcPHB* among lobed accessions (*n* = 63) and entire accessions (*n* = 194). **B ***π* values associated with *BcPHB* in lobed and entire accessions. **C** Model of BcLMI1-BcPHB regulation relationship in leaf lobe formation in *B. campestris*

*B. campestris* was domesticated from its wild relative in China ~ 7000 years ago. Since leaf margin significantly influences yield, it has been a crucial factor in *B. campestris* breeding. In *B. campestris*, the leaves of relatively primitive populations are usually lobed, such as those of the turnip, which is distributed in northwestern China. Entire-leaved varieties emerged relatively later and are mainly distributed along the southeastern coastal regions of China, such as *pak-choi*, and in southern China, such as *caixin* (Cai et al. 2021). After investigating resequenced *B. campestris* accessions, including different accessions with wide distribution in China, we observed that 63.49% of the accessions, which exhibit lobed leaves, were characterized by the presence of *BcPHB*^*HapII*^. However, this percentage decreased to 9.28% in accessions with entire leaves (Fig. 7A). Consequently, the proportion of *B. campestris* with entire leaves, characterized by the presence of *BcPHB*^*HapI*^, increased during the domestication. *π* analyses also revealed that lobed accessions exhibited higher diversity than entire accessions, indicating preferential artificial selection for *BcPHB* during *B. campestris* domestication (Fig. 7B). The result suggested the two haplotypes of *BcPHB* have been utilized in *B. campestris* breeding in different regions, but have not been fully fixed.

## Discussion

Dissecting the genetic basis of complex morphological traits like leaf lobation presents a dual challenge: accurately quantifying the phenotype and identifying the underlying molecular pathways. In this study, we addressed both aspects in *B. campestris*. Methodologically, we established a pipeline for the precise and high-throughput quantification of leaf lobation. Our approach, which produces a composite index of six biologically interpretable geometric variables, provides a robust solution (Fig. 1). Unlike abstract methods like elliptic Fourier descriptors, these variables correspond directly to the degree of marginal lobes, thus enhancing biological interpretability. This high-throughput, objective phenotyping platform is not limited to *Brassica*; it provides a transferable methodology for dissecting complex leaf shape variation in other important crops where such diversity is prevalent, including cruciferous vegetables such as mustard, composite crops like lettuce, and various woody plants.

On the molecular level, our work addresses a long-standing gap: the upstream regulation of the conserved LMI1 pathway. While LMI1 is unequivocally a pivotal factor for leaf margin patterning across diverse species, its upstream developmental modulators have remained largely elusive. In our parallel genetic investigation, we identified the adaxial-abaxial polarity gene *BcPHB* as one of these critical, long-sought regulators on leaf margins (Fig. 2A, B). As a known regulator of adaxial-abaxial polarity (Emery et al. 2003), the promoter variation of *BcPHB* was stably associated with lobation depth: high-expression haplotype conferred entire margins, whereas low-expression haplotype promoted lobes (Fig. 2D-F). This extends the role of PHB beyond its classic function in polarity establishment to that of a quantitative modulator of marginal growth. *Cis*-regulatory changes frequently underlie continuous phenotypic variation (Wray 2007), and our results highlight promoter variation as a key mechanism shaping leaf margin complexity.

 Our transactivation assays in yeast showed that BcPHB possesses intrinsic transcriptional activation potential (Fig. 3D), whereas *in planta* assays demonstrated that BcPHB acts as a repressor of *BcLMI1* (Fig. 4D). This discrepancy is likely due to the limitations of the heterologous yeast system, which lacks the specific chromatin landscape or the complex native regulatory environment present in plant cells. Similar phenomena have been reported for other transcription factors; for instance, the *Medicago* transcription factor MtMYB3 displays transcriptional activation activity in yeast but functions as a direct repressor of *MtCBF4* in plants (Zhang et al. 2016). Thus, in its native context, BcPHB likely functions within a repressive framework that cannot be fully recapitulated in yeast. BcPHB and BcLMI1 form a reciprocal inhibitory loop (Fig. 7C), this circuit may operate as a bistable switch, converting graded molecular inputs into two stable outputs: high PHB/low LMI1 (entire leaves) and low PHB/high LMI1 (lobed leaves). Such bistable systems are well-suited for boundary patterning, partitioning uniform cell fields into distinct developmental fates. Compared with the strong-effect LMI1/RCO pathway, which drives large categorical shifts in leaf shape and organ proportions (Streubel et al. 2018; Vlad et al. 2014a), *PHB* promoter variation acts as a fine-scale tuner. This explains the continuous spectrum of lobation observed within *B. campestris* and complements the function of major regulators. Our results extend the established role of HD-ZIP III transcription factors in leaf polarity and margin formation in Arabidopsis and *C. hirsuta* to a domesticated *Brassica* species.

Our findings also highlight the impact of domestication on leaf morphology. Lobed leaves (*BcPHB*^*HapII*^) are common in primitive populations, whereas entire leaves (*BcPHB*^*HapI*^) dominate in cultivated accessions (Fig. 7A). This pattern indicates artificial selection on *BcPHB*, consistent with broader trends in crop domestication where leaf shapes were optimized for yield, marketability, and biomass allocation. By contrast, human selection prioritized uniform biomass distribution and ease of harvest, favoring entire-leaf phenotypes. The lobed-to-entire transition, therefore, reflects cultivation-driven adaptation rather than inherent evolutionary superiority.

Beyond morphological remodeling, *BcPHB* suppression/BcLMI1 overexpression was associated with elevated net photosynthesis and transpiration rates (Figs. 3H, I and 5I, J). The mechanistic basis of this physiological shift likely involves multiple contributing factors. The disorganized mesophyll architecture observed in *BcPHB*^*ami*^ and *BcLMI1*^*OE*^ leaves (Figs. 3G and 5H) may increase intercellular air spaces and enhance mesophyll conductance (*g*_m_), facilitating CO₂ supply to chloroplasts (Flexas et al. 2008). Additionally, consistent with prior reports that lobed leaf margins reduce aerodynamic boundary layer resistance and improve gas exchange efficiency (Vogel 2009), the lobed geometry itself may also contribute. Distinguishing the relative contributions of these external and internal structural factors will require future work, including direct *g*_m_ measurements and experiments that decouple lobing from mesophyll reorganization.

Together, the establishment of a reproducible quantitative phenotyping system and our discovery of BcPHB as the upstream regulator of *BcLMI1* delineate a complex regulatory landscape underlying lobation. These findings provide mechanistic insights into both developmental regulation and domestication-driven evolution in *B. campestris*. Looking forward, advancing from correlation to causality will require developmental time-series phenotyping and multi-angle imaging (Murphy et al. 2024), as well as causal tests of promoter variants using reporter assays, transcription factor occupancy, and base editing (Wray 2007). Such efforts will clarify the dose–response relationship between *PHB* expression and margin morphology, and enable controlled tuning of lobation in leafy crops.

This study introduces a framework for the highly accurate and objective profiling of leaf lobation, setting a standard for quantitative analysis. Concurrently, we answer a fundamental question in plant developmental biology by discovering the *B. campestris* ortholog *BcPHB* as a key orchestrator upstream of the conserved leaf-margin regulator *LMI1.* We elucidate a novel reciprocal negative feedback loop wherein BcPHB and BcLMI1 collectively govern leaf margin development. Crucially, our research forges a clear mechanistic connection between this developmental module and crop evolution, demonstrating how artificial selection acting on *cis*-regulatory variation in the *BcPHB* promoter sculpted the diverse leaf architectures observed in *B. campestris*. Together, these findings not only provide a deep mechanistic understanding of complex organ development but also offer versatile genetic targets for engineering optimized plant architecture in a wide range of crop species.

## Materials and methods

### Precise phenotypic identification

*B. campestris* accessions were cultivated at the experimental base of Zhejiang University, Hangzhou, China. At the normal harvest stage, representative plants from each accession were selected, and their largest rosette leaves were detached and photographed. Each image contained a 6 cm^2^ black-and-white calibration block and a sample label for color and scale correction.

According to the *Descriptors and Data Standard for Chinese Cabbage Germplasm Resources*, a digital phenotyping pipeline was developed to quantitatively describe margin types. Leaf contours were extracted, and the upper half of each leaf margin was fitted with an ellipse, which served as a reference baseline for calculating vertical distances to detect peaks and valleys. Based on expert visual classification, leaf images were categorized into three classes (level 1, level 2, and level 3) to construct a labeled dataset.

To comprehensively compare the five algorithms, six complementary metrics were computed under stratified fivefold cross-validation and arranged along the axes of a radar chart: accuracy (overall fraction correct), macro-Precision and macro-Recall (unweighted per-class means of TP/(TP + FP) and TP/(TP + FN), giving each class equal weight), macro-F1 (their harmonic mean), ROC AUC one-vs-rest (a threshold-independent measure of ranking quality), and Cohen’s κ (chance-corrected agreement with the expert annotations).

All data processing and model training were implemented in Python using OpenCV (for contour extraction) and scikit-learn (for classification). The six contour-derived features were standardized (zero mean, unit variance) before model fitting. The five classifiers, KNN (*k* = 5), Naïve Bayes, random forest (200 trees), SVM (RBF kernel, C = 10, *γ* = “scale”), and a multilayer perceptron (two hidden layers of 32 and 16 units), were evaluated under stratified fivefold cross-validation (random_state = 42).

### Data processing

Sequencing data were generated using the Illumina NovaSeq6000 platform (Biomarker). Low-quality reads were filtered by using fastP (Chen et al. 2018) with default parameters. Filtered reads were then mapped to the *B. campestris* reference genome v3.5 (Chen et al. 2022; Zhang et al. 2022) using BWA-mem (Li 2013). Mapping results were sorted and marked duplicates using PICARD. GATK4.0 HaplotypeCaller (McKenna et al. 2010) was used for the identification of SNPs and InDels, following its default parameters. Subsequently, raw variations of low quality were filtered out.

### GWAS analysis for the leaf margin trait

A total of 1,583,963 SNPs from 291 *B. campestris* accessions were used to perform GWAS for the leaf lobe trait. Only SNPs with an MAF > 0.05 and a missing rate < 0.1 in the population were used in the GWAS. An association analysis was performed by a mixed linear model (MLM) with the population structure and kinship implemented in the GEMMA software package (Zhou and Stephens 2012). The Manhattan and quantile–quantile plots were generated using the R package *qqman* (Turner 2018, 2014).

### Selective sweep analysis for the leaf margin trait

Selective-sweep analysis was carried out between accessions with entire leaves and those with lobed leaves to further search for the loci associated with the leaf margin trait on the genome. The population differentiation index (*F*_ST_) was computed using the VCFtools (Danecek et al. 2011). The top 1% of *F*_ST_ values were screened as the selected regions, with a window size of 50 kb and a step size of 5 kb.

### RT-qPCR

Leaf samples from different cultivars and transgenic plants grown for 20 days after sowing (DAS) were taken for total RNA isolation using Trizol (CAT#: AG21102, Accurate Biology, Changsha, China), and the first-strand cDNA was synthesized using 1 μg total RNA by HiScript III RT SuperMIX for qPCR (CAT#: R323-01, Vazyme, Nanjing, China). RT-qPCR was performed using SYBR Green Pro Taq HS (CAT#: AG11701, Accurate Biology, Changsha, China). The gene-specific primers used in this research are detailed in Table S7.

### Promoter activity analysis

To verify the transcriptional activity differences between the two haplotypes, the promoter regions of *BcPHB* from HapI (entire-leaf accession) and HapII (lobed-leaf accession) were isolated. Approximately 1,000 bp upstream of the translation start site was amplified using specific primers (Table S7). The PCR products were cloned into the pGreenII 0800-LUC vector to generate the reporter constructs. The Renilla luciferase (REN) gene driven by the CaMV 35S promoter served as an internal control. The constructs were transformed into *Agrobacterium tumefaciens* strain GV3101 (pSoup-p19) (CAT#: AC1003, WeiDi, Shanghai, China). Positive colonies were resuspended in infiltration buffer (10 mM MES, 10 mM MgCl2, and 150 μM acetosyringone) to a final OD_600_ of 0.8 and incubated at room temperature for 3 h. The suspensions were then infiltrated into young leaves of 4-week-old *Nicotiana benthamiana* plants. After 48 h of post-infiltration growth, leaf discs were harvested. The LUC and REN activities were measured using the Dual-Luciferase Reporter Assay System (Promega, USA) according to the manufacturer’s instructions. The relative promoter activity was calculated as the ratio of LUC to REN.

### Vector construction and plant transformation

The amiRNA of the *BcPHB* gene was designed based on the *MIR164* backbone and synthesized by Beijing Tsingke Biotech Co., Ltd. (Beijing, China), then cloned into the pFGC1008-HA plasmid. The full-length fragment of the *BcPHB* gene driven by the self-promoter of *BcPHB*^*HapI*^ was constructed onto the pCAMBIA1300 vector. The coding sequences (CDS) of *BcLMI1* were amplified and subsequently cloned into the pFGC1008-HA vector. These constructions were delivered to *A. tumefaciens* strain GV3101 (CAT#: AC1001, WeiDi, Shanghai, China) and then transformed into *B. campestris* plants through the floral dip method (Gao et al. 2012). Genomic PCR was used to identify and validate transgenic plants.

### LMI1 evolutionary conservation analysis

The protein sequences of *B. campestris* were obtained from the Brassica Database (BRAD; http://www.brassicadb.cn) (Chen et al. 2022). LMI1 sequences of *Vitis vinifera* (Vvinifera_145), *Selaginella moellendorffii* (Smoellendorffii_91_v1.0), *Populus trichocarpa* (Ptrichocarpa_533_v4.1), *Carica papaya* (Cpapaya_113_ASGPB_v0.4), *Volvox carteri* (Vcarteri_317_v2.1), *Amborella trichopoda* (Atrichopoda_291_v1.0), and *Physcomitrella patens* (Ppatens_318_v3.3) were downloaded from Phytozome v13 (Goodstein et al. 2012). LMI1 sequences from other species were obtained from the PlantTFDB v4.0 (Jin et al. 2017). 48 HD-zip protein sequences from *A. thaliana* were downloaded from TAIR (https://www.arabidopsis.org/) (Garcia-Hernandez et al. 2002) and used as query sequences to identify putative *HD-ZIP* genes in *B. campestris* via BLAST search (E-value < 1e − 5) using TBtools v2.0 (Chen et al. 2020; 2023). The identified sequences were validated using the Conserved Domain Database (CDD; http://www.ncbi.nlm.nih.gov/cdd/) (Lu et al. 2020) with an E-value threshold of 0.01, and the Pfam database (http://pfam.xfam.org/) (Punta et al. 2012) with default parameters. Non-redundant sequences were retained and designated as *HD-ZIP* genes. Multiple sequence alignments were performed using MUSCLE (Edgar 2004a, 2004b) implemented in MEGA v11.0 software (Tamura et al. 2021). Phylogenetic trees were constructed using IQ-TREE v2.1 (Minh et al. 2020) with 1000 bootstrap replicates.

### GUS histochemical staining assay

To create the fusion constructs *BcPHBpro::GUS* and *BcLMI1pro::GUS*, the upstream ~1000 bp fragments of *BcPHB* and *BcLMI1 *were amplified and introduced into the pCAMBIA1300-GUS vector. These constructs were delivered to *A. tumefaciens* strain GV3101 (CAT#: AC1001, WeiDi, Shanghai, China) and then transformed into *B. campestris* plants through the floral dip method (Gao et al. 2012). Genomic PCR was used to identify and validate transgenic plants. The first true leaves of the *BcPHBpro::GUS* and *BcLMI1pro::GUS* transgenic plants were collected at 7 DAS and stained in GUS staining buffer (CAT#: SL7160, Coolaber, Beijing, China). Subsequently, the samples were bleached with 75% ethanol and photographed.

### Subcellular localization

To create the fusion constructs *35S::BcPHB-eGFP* and *35S::BcLMI1-eGFP*, the CDS of *BcPHB* and *BcLMI1* were amplified and introduced into the pFGC-eGFP vector. The fusion constructs were employed for transient gene expression investigation in *N. benthamiana* leaves, together with an H2B-RFP as a nucleus-localized marker. As a negative control, construct *35S::eGFP* was employed. GFP fluorescence was observed two days after agroinfiltration using TCS SP8-SE (Leica, Wetzlar, Germany) with a 488 nm excitation wavelength and a 505–530 nm bandpass emission filter.

### Transcriptional activity verification

The pGBKT7-BcPHB (BD-PHB) and pGBKT7-BcLMI1 (BD-BcLMI1) were transformed into Y2H Gold (CAT#: YC1002, WeiDi, Shanghai, China), respectively. The plasmid pGBKT7 served as a negative control. Transformants were cultured on SD/-Trp medium (CAT#: PM2252, Coolaber, Beijing, China). Positive colonies were then transferred to SD/-Trp/-His medium (CAT#: PM2282, Coolaber, Beijing, China) and incubated at 30 °C for 72 h.

### SEM

The epidermal morphology of the 35-day-old leaves was observed by SEM. Leaf samples were processed according to previously published procedures (Wan et al. 2021). The samples were observed in a Hitachi Model SU-8010 SEM (Tokyo, Japan).

### Paraffin section

The 35-day-old leaves were collected and then fixed in the 70% FAA solution (CAT#: G1103-500ML, Servicebio, Wuhan, China). Following fixation, the samples underwent a meticulous dehydration process, which involved a gradual increase in ethanol (CAT#: 64–17-5, Hushi, Shanghai, China) concentration, starting from 70% and progressing to 100%. After dehydration, the samples were cleared in xylene (CAT#: 1330–20-7, Hushi, Shanghai, China) and then embedded in paraplast (CAT#: 39,601,006, Leica, Germany). The tissues were sectioned to 10 μm. Slides were dewaxed and stained with tolonium chloride stain (CAT#: T104232, Aladdin, Shanghai, China).

### Determination of the net photosynthesis rate and the transpiration rate

Plants were planted at 22/18 °C with a 16-h light and 8-h dark cycle. TP-PM-1 plant photosynthetic apparatus (Topyunnong Technology Co., Ltd.) was used for the determination of physiological parameters. The specifications of the leaf chamber were 3 cm^2^. The net photosynthesis rate and transpiration rate measurement resolutions were 0.001. Each plant was measured three times.

### Y1H assay

The promoter fragments of *BcPHB* and *BcLMI1* were respectively fused with the pLacZi (Promega, Madison, WI, USA) vector as baits, while the CDS of *BcPHB* and *BcLMI1* were fused with the pB42AD as prey, respectively. The bait and prey plasmids were co-transferred into EGY48 (CAT#: YC1030, WeiDi, Shanghai, China) and screened on SD/-Trp/-Ura medium (CAT#: PM2260, Coolaber, Beijing, China) and SD/-Trp/-Ura dropout medium containing X-gal (CAT#: CX11921, Coolaber, Beijing, China) at 30 °C. Empty pB42AD served as the negative control.

### ChIP-qPCR

To create the fusion constructs *35S::BcPHB-HA* and *35S::BcLMI1-HA*, the CDS of *BcPHB* and *BcLMI1* were amplified and introduced into the pFGC1008-HA vector. These constructions were delivered to *A. tumefaciens* strain GV3101 (CAT#: AC1001, WeiDi, Shanghai, China) and then transformed into *B. campestris* plants through the floral dip method (Gao et al. 2012). Genomic PCR was used to identify and validate transgenic plants. The leaves of 35-day-old *35S::BcPHB*^*OE*^*-HA* and *35S::BcLMI1*^*OE*^*-HA* transgenic plants were used and were sampled. Total proteins were extracted from leaf tissues using an extraction buffer (50 mM Tris–HCl, pH 7.5, 150 mM NaCl, 1 mM EDTA, 10% glycerol, 1 mM PMSF, and 1 × protease inhibitor cocktail). The extracts were centrifuged at 12,000 rpm for 10 min at 4 °C, and the supernatants were separated on 10% SDS-PAGE gels and transferred to PVDF membranes. The membranes were blocked with 5% skim milk and incubated with anti-HA (HA-Tag (C29F4) Rabbit mAb, Cell Signaling Technology, Danvers, MA, USA), followed by HRP-conjugated secondary antibody. Signals were detected using a chemiluminescence kit (Tanon 5200, Shanghai, China). The ChIP assay was carried out using the EpiQuik Plant ChIP Kit (CAT#: P-2014–24, Epigentek, Farmingdale, USA). Primers were designed based on the predicted binding motifs by using JASPAR (Khan et al. 2018) (Table S7). RT-qPCR was conducted using SYBR Green Pro Taq HS (CAT#: AG11701, Accurate Biology, Changsha, China).

### Luciferase bioluminescence imaging assay and DLR assay

To identify the functional SNPs responsible for the differential expression of *BcPHB*, five specific reporter constructs were prepared: the natural *proBcPHB*^*HapI*^ (high-activity), the natural *proBcPHB*^*HapII*^ (low-activity), a reverted version of HapII (*proBcPHB*^*HapII*^*-rev*) where the target SNPs were changed to the HapI sequence, a reciprocal mutated version of HapI (*proBcPHB*^*HapI*^*-mut*) where the target SNPs were changed to the HapII sequence, and an empty pGreenII 0800-LUC vector as a negative control.

The CDS of *BcPHB* and *BcLMI1* were fused with the pGreenII 62-SK vector, respectively (Promega, Madison, WI, USA). The promoter fragments of *BcPHB* and *BcLMI1* were fused with pGreenII 0800-LUC (Promega, Madison, WI, USA), respectively. Subsequently, all the constructs were co-transformed into the GV3101 (pSoup-p19) Chemically Competent Cell (CAT#: AC1003, WeiDi, Shanghai, China). The pGreenII 62-SK empty vector was used as a negative control. *A. tumefaciens* carrying the effector and reporter constructs was used to infect the same *N. benthamiana* leaf in the dark at 25 °C for 36 h. The D-Luciferin potassium salt (CAT#: ST196, Beyotime, Shanghai, China) was used in the luciferase bioluminescence imaging assay. The activated luciferase reconstitution signals were imaged using the Tanon 5200 chemiluminescence image analysis system (Shanghai, China). DLR assays were conducted using the Dual-Luciferase Reporter Assay System (CAT#: E1910, Promega, Madison, USA).

### Nucleotide diversity analysis

Genomic variants within a 0.5 Mb region flanking the *BcPHB* gene were extracted using BCFtools (v1.10.2) (Danecek et al. 2021). Subsequently, nucleotide diversity (*π*) across different populations was calculated using VCFtools (v.0.1.16) (Danecek et al. 2011) with a sliding window of 50 kb and a step size of 5 kb.

### Statistical analysis

Statistical differences between groups were determined using GraphPad Prism version 10.

## Supplementary Information

Supplementary Material 1. Figure S1 Performance comparison of five machine learning classifiers for leaf margin level classification. Figure S2 Quantile–quantile plots of genome-wide association study (GWAS). Figure S3 Functional validation of the causal SNPs in the *BcPHB* promoter. Figure S4 Expression level of *BcPHB* in leaves at different leaf positions of *Brassica campestris*. Figure S5 Expression level of *BcPHB* in *BcPHB*^*ami*^ transgenic plants. Figure S6 Quantification of *BcPHB* transcript levels in complementation lines. Figure S7 Phylogenetic tree of HD-ZIP genes in *Brassica campestris*. Figure S8 Phylogenetic relationships and conserved domain organization of LMI1. Figure S9 Expression analysis of *BcLMI1* in different *B. campestris* accessions. Figure S10 Western blot of *BcPHB*^*OE*^-3HA tag lines and *BcLMI1*^*OE*^-3HA tag lines. Figure S11 Expression level of *BcLMI1* in *BcLMI1*^*OE*^ transgenic plants.

Supplementary Material 2. Table S1 Six contour-derived indicators for quantifying leaf margin lobation. Table S2 Leaf margin trait-related genes identified by genome-wide association study (GWAS). Table S3 Leaf margin trait-related genes identified by selective sweep analysis. Table S4 Regions of linkage disequilibrium (LD) blocks. Table S5 Annotation of genes within association intervals. Table S6 SNPs in the promoter and exonic regions of *BcPHB*. Table S7 Primers used in this study.

## Data Availability

The sequencing data have been deposited in the National Center for Biotechnology Information BioProject database under accession PRJNA1072754. The reviewer link is https://dataview.ncbi.nlm.nih.gov/object/PRJNA1072754?reviewer=b5f0shk921snusigu611qtl6th.

## Abbreviations

35S: Cauliflower mosaic virus 35S promoter
ami: Artificial microRNA
ANOVA: Analysis of variance
*B. campestris*: *Brassica campestris*
*B. rapa*: *Brassica rapa*
*BcLMI1*: *Brassica campestris LATE MERISTEM IDENTITY 1*
*BcPHB*: *Brassica campestris PHABULOSA*
CDS: Coding sequences
ChIP-qPCR: Chromatin immunoprecipitation quantitative PCR
NN: Neural network
DAS: Days after sowing
DLR: Dual-luciferase reporter
eGFP: Enhanced green fluorescent protein
*F*_ST_: Population differentiation index
FAA: Formalin-Aceto-Alcohol solution
FPKM: Fragments per kilobase per million mapped fragments
GUS: *β*-Glucuronidase
GWAS: Genome-wide association study
HA: Hemagglutinin tag
Hap: Haplotype
HD-ZIP: Homeodomain-leucine zipper
HRP: Horseradish peroxidase
IPA: Ideal plant architecture
ALH: Average lobing height
MLH: Maximum lobing height
NPV: Number of peaks and valleys
MPH: Maximum peak height
TLH: Total lobing height
MVD: Maximum valley depth
LUC: Firefly luciferase
MAF: Minor allele frequency
NC: Negative control
OE: Overexpression
*π*: Nucleotide diversity
PCA: Principal component analysis
PVDF: Polyvinylidene fluoride
REN: Renilla luciferase
RFP: Red fluorescent protein
SD: Standard deviation
SDS-PAGE: Sodium dodecyl sulfate–polyacrylamide gel electrophoresis
SEM: Scanning electron microscopy
SNP: Single-nucleotide polymorphism
Y1H: Yeast one-hybrid
