# Supplementary material for: Machine learning-guided leaf margin classification and discovery that BcPHB promoter selection rewires a BcPHB–BcLMI1 module to shape leaf margins in Brassica campestris
Source: Mol Hortic. 2026 Oct 13;6:75. doi: 10.1186/s43897-026-00251-w (PMC13660319; doi:10.1186/s43897-026-00251-w)
Supplement: Supplementary file 1 [file 43897_2026_251_MOESM1_ESM.docx]

**Figure S1** **Performance comparison of five machine learning classifiers for leaf margin level classification.** The radar chart displays six evaluation metrics (accuracy, macro-Precision, macro-Recall, macro-F1, ROC AUC one-vs-rest, and Cohen’s κ) computed under stratified 5-fold cross-validation for K-Nearest Neighbor (KNN), Naïve Bayes (NB), Random Forest (RF), Support Vector Machine (SVM), and a neural network (NN); larger polygon area indicates better overall classification quality.

**Figure S2 Quantile–quantile plots of genome-wide association study (GWAS).** The x-axis represents expected -log_10_ (*P-*value), and the y-axis represents observed -log_10_ (*P-*value).

**Figure S3 Functional validation of the causal SNPs in the *BcPHB* promoter.** **(A)** Representative images of the dual-luciferase reporter assay in *Nicotiana benthamiana* leaves. The infiltration positions (1–5) correspond to the following constructs: 1, *proBcPHB^HapI^-LUC*; 2, *proBcPHB^HapII^-rev-LUC* (reverted version); 3, *proBcPHB^HapI^-mut-LUC* (mutated version); 4, *proBcPHB^HapII^-LUC*; 5, Empty vector (negative control). The pseudo-color scale represents the intensity of the luminescence signal. **(B)** Relative LUC/REN activity of the different promoter constructs. Data represent the mean ± SD of five biological replicates (*n* = 5). Different letters above the bars indicate significant differences (*P* < 0.05) according to Tukey’s multiple comparisons test.

**Figure S4 Expression level of *BcPHB* in leaves at different leaf positions of *Brassica campestris*.** 1-6 represents the first to the sixth true leaves. The error bar represents the mean with SDs.

**Figure S5 Expression level of *BcPHB* in *BcPHB^ami^* transgenic plants.** The results are shown as the mean ± SDs. RT-qPCR data were normalized using *BcUBC10*. One-way ANOVA Dunnett’s multiple comparison test, ***P* < 0.01. Ami: artificial miRNA.

**Figure S6 Quantification of *BcPHB* transcript levels in complementation lines.** Relative expression of *BcPHB* determined by RT-qPCR in the natural entire-leaf accession (YQ49), the lobed-leaf accession (WZPC), and two independent complementation lines (*proBcPHB^HapI^::BcPHB*-1 and -2). Data represent the mean ± SD. Different letters above the bars indicate significant differences (*P* < 0.05) based on One-way ANOVA followed by Tukey’s multiple comparisons test.

**Figure S7 Phylogenetic tree of HD-ZIP genes in *Brassica campestris*.** A total of 81 HD-zip genes in *B. campestris* were identified, respectively. *BcPHB* and *BcLMI1* identified in this study were marked in bold red font.

**Figure S8 Phylogenetic relationships and conserved domain organization of LMI1.** A phylogenetic tree was constructed based on LMI1 protein sequences identified from representative plant species. *Selaginella moellendorffii*, *Volvox carteri*, and *Physcomitrella patens* were searched but yielded no significant LMI1 homologs and are therefore not represented in the tree. The left panel shows the evolutionary relationships among LMI1s, with bootstrap values indicated at the branch nodes. The right panel shows the corresponding domain organization of each protein, where the Homeodomain is shown in blue, the HALZ domain is shown in pink, and gray regions represent protein sequences. The numbers -1, -2, -3, and -4 denote different copies within a single species.

**Figure S9 Expression analysis of *BcLMI1* in different *B. campestris* accessions.** Relative transcript levels of *BcLMI1* in the four lobed-leaf accessions (S1–S4) and four entire-leaf accessions (S5–S8). Data represent mean ± SD (*n* = 3).

**Figure S10 Western blot of *BcPHB^OE^-3HA* tag lines and *BcLMI1^OE^-3HA* tag lines.**

Abundance of *BcPHB^OE^-3HA* and *BcLMI1^OE^-3HA* in the leaves of T_2_ transgenic plants, as determined by immunoblotting using anti-HA antibodies.

**Figure S11 Expression level of *BcLMI1* in *BcLMI1^OE^* transgenic plants.** The results are shown as the mean ± SDs. RT-qPCR data were normalized using *BcUBC10*. One-way ANOVA Dunnett’s multiple comparison test, ***P* < 0.01.
